# Process evaluation of the Belgian one-month-without alcohol campaign ‘Tournée Minérale’: a mixed method approach

**DOI:** 10.1186/s12889-024-17941-z

**Published:** 2024-02-05

**Authors:** Annelies Thienpondt, Jelle Van Cauwenberg, Joris Van Damme, Benedicte Deforche

**Affiliations:** 1 Department of Public Health and Primary Care, Ghent University, Corneel Heymanslaan 10, Ghent, B- 9000 Belgium; 2 Research Foundation Flanders (FWO), Brussels, Belgium; 3 Flemish expertise centre on alcohol and other drugs, Vanderlindestraat 15, Schaarbeek, 1030 Belgium; 4 Movement and Nutrition for Health and Performance Research Unit, Faculty of Physical Education and Physical Therapy, Vrije Universiteit Brussel, Pleinlaan 2, Brussels, B-1050 Belgium

**Keywords:** Alcohol use, Mass media campaign, Adult, Temporary abstinence, Refraining from alcohol, Prevention, Intervention

## Abstract

**Background:**

The Tournée Minérale campaign [TMC] is a mass media prevention campaign challenging Belgian adults to refrain from alcohol during one month. A process evaluation may help us better understand the effect of TMC and to formulate recommendations for future editions. The current study aimed to examine reach, experiences, perceived effectiveness and maintenance of TMC.

**Methods:**

A mixed method design was used to assess the process, using pre- and post-questionnaires (*n* = 49.022, 44.5 ± 13.1 years old, 37.0% men) and focus groups (*n* = 31, 47.3 ± 14.3 years old, 33.3% men).

**Results:**

Most campaign materials were considered useful and/or motivating. Facilitators for taking part in TMC were connectedness with other participants, stimulus control (e.g. removing alcohol at home) and a supportive social environment. Most difficulties were encountered with abstaining during social occasions as participants had to change a habit or find alternative non-alcoholic beverages. Participants reported both beneficial (e.g. sleeping better) and adverse effects (e.g. drinking more soft drinks).

**Conclusions:**

Future editions of TMC should try to decrease perceived adverse effects (e.g. by providing attractive non-alcoholic beverages) and can benefit from having a forum where people can share experiences.

**Supplementary Information:**

The online version contains supplementary material available at 10.1186/s12889-024-17941-z.

## Background

Harmful alcohol use is responsible for 5.1% of the global burden of disease [1] and has negative social consequences (e.g. alcohol-related crime, violence and injuries) [2, 3]. To tackle the impact of alcohol consumption, public health interventions such as a temporary abstinence campaign [TAC] are promising. TACs challenge adults to refrain from alcohol during a fixed period (often one month) and several effect evaluations show reductions in alcohol consumption after participation [4, 5]. However, effect evaluations [4–8] only offer limited understanding of the underlying mechanisms of the intervention effects. Process evaluations on the other hand provide such insights and deliver valuable information for improving future TACs [9].

There is no single definition of a process evaluation [10] and different process evaluation frameworks exist [11–14] such as the RE-AIM framework [15], the process evaluation elements of Saunders [14] and Grant’s process evaluation framework [9]. To provide a comprehensive understanding of TACs’ underlying mechanisms from a participant’s perspective, it is important to combine different frameworks since each framework has its limitations. For example, the RE-AIM framework does not include the recruitment process or unintended consequences due to the intervention (i.e. beneficial or harmful outcomes) from a participant’s perspective. Saunders’ framework lacks the effectiveness and maintenance elements of a process evaluation, and Grant’s framework does not assess the experiences of the participants. Therefore, for the current study we combined the three frameworks and identified four key elements to provide a comprehensive process evaluation of a TAC. The first key element is *“reach” (*based on ‘reach’ in RE-AIM [15], Saunders [14] and Grant [9], with the addition of Grant’s ‘recruitment’ element [9]), i.e. channels to reach participants, representativeness of participants, motivations and reasons for (not) participating. The second key element is *“experiences”* (based on the individual component of RE-AIM’s ‘implementation’ [15] and Saunder’s ‘context’ [14]), i.e. participants’ use and response to the intervention (materials). The third key element is “*effectiveness*” (based on ‘effectiveness’ of RE-AIM [15] & Grant [9] and Grant’s ‘unintended consequences’ [9]), i.e. perceived impacts of the intervention (both desired and adverse effects). The fourth and last key element is “*maintenance*” (based on ‘maintenance’ of RE-AIM [15] & Grant [9]), i.e. perceived maintenance of behavior change after the intervention. The current study focused on participants’ experiences, while the experiences of implementers and adopters (such as the ‘adoption’ element in RE-AIM, Saunder’s ‘dose-delivered’ or Grant’s ‘delivery’) were beyond the scope of the current study. Previous process evaluation studies of TACs have focused on some (parts of) key elements such as the motivations for participating (part of *reach*), the evaluation of (some) campaign materials (part of *experiences*) and perceived beneficial effects of participating (part of *effectiveness*) [16–19]. Other subcomponents of TAC evaluations i.e. the channels to reach participants, representativeness of participants and reasons for not participating (*reach*); participants’ use and response to all intervention components and materials (*experiences*); perceived impact of the intervention (including both desired and adverse effects) (*effectiveness*); and perceived maintenance of behavior change after the intervention (*maintenance*), have not yet been studied in depth. All above mentioned key elements are examined in the current study.

The effect evaluation of The Tournée Minérale campaign [TMC] (TAC in Belgium) showed a decrease among TMC participants in weekly alcohol consumption six weeks (-30%) and six months after participation (-19%). Furthermore, a reduction in the proportion of excessive weekly users and binge drinkers and changes in several underlying determinants of alcohol consumption were found among TMC participants [8].

To obtain more detailed insights about the findings of this effect evaluation, the current paper aims to examine different subcomponents of reach, experiences, perceived effectiveness and maintenance of TMC. This may help us better understand the mechanisms behind the effects (not) observed in the effect evaluation study and enables us to formulate recommendations for future TACs.

## Methods

### Intervention: TMC

The national Foundation against Cancer and “de druglijn”, a Flemish government subsidized service which provides information and help about substance use, developed and funded the first TMC in 2017. The aim was to help Belgian adults to reduce their alcohol consumption by raising awareness about (excessive) alcohol consumption and to raise money for cancer research by urging them not to drink alcohol during the month February. All Belgian adults were encouraged to participate by a large-scale nationwide campaign using promotional videos, advertisements, banners and social media. They were also encouraged to visit the TMC website (https://www.tourneeminerale.be/) where they could register to participate. An overview of campaign materials can be found in Supplementary File 1.

### Study design and context

This study is part of a mixed methods design with pre- and post-measurements that investigated the effect of TMC [8]. To answer the current research questions, we used quantitative as well as qualitative research methods to obtain detailed and rich information [20]. Quantitative questionnaires were used to assess key aspects of the process of TMC and qualitative questions (i.e. focus group interviews) were designed to elicit in-depth information and ask questions that could not be quantified. Quantitative and qualitative data were combined to provide a detailed and comprehensive process evaluation of TMC. To improve the readability of this manuscript, we linked the content of the quantitative data to data from the focus groups. Quantitative questionnaires were completed before and after TMC by participants. In addition, focus groups with participants of TMC were held after TMC. For the quantitative study, all adults (≥ 18 years) who understood the purpose and content of the study and signed the online informed consent form were eligible to participate. For the qualitative study, participants indicated before the start of the focus groups that they participated voluntarily and gave permission to record the interview with an audio recorder by signing the informed consent form. The researchers clearly communicated that the recordings would only be listened to for the purpose of analyzing the focus group and that they would be discarded at the end of the study. Furthermore, any material that could potentially identify a participant was treated with caution and subsequently destroyed. The researchers also emphasized that everyone’s ideas were important and that there were no wrong ideas. The researchers remained independent from the intervention developers. The recruitment process explicitly stated that the questionnaire and focus group study was from Ghent University and not TMC’s developers. The study was approved by the ethical committee of the Ghent University Hospital (2017/0069).

### Sampling and data collection

For the quantitative part of the study, all registered participants on the TMC website were invited through e-mail to complete an online baseline questionnaire. Those who completed the baseline measurements were contacted for post measurements through e-mail. The measurements took place in January (baseline) and March (post) 2017, with February as intervention period (TMC). A reminder was sent twice to those who had not yet responded. A total of 123,842 persons registered on the campaign website, of which 49,022 people completed the baseline questionnaire and 16,547 completed the post measurements. Compared to the general Belgian population, participants of TMC were older, more likely to be female and higher educated but had a similar weekly alcohol consumption [8].

For the qualitative part of the study, focus groups were held with TMC participants. The focus groups were held in Dutch (the researcher’s native language) and were conducted in the five Dutch speaking Belgian provinces (Antwerp, Limburg, East Flanders, Flemish Brabant and West Flanders). One township or city was selected by convenience in each of the provinces. Based on the postal code asked at baseline measurements, TMC participants from each selected township/city were invited by e-mail for the focus groups. Additional participants were recruited through a snow ball sampling method performed by enrolled participants and via master students, who helped the researcher during the study. The focus groups were conducted between March 16 and April 20, 2017 in a neutral room at a central location in the selected city/township (maximum twenty minutes by car for each participant). The duration of the interviews ranged from 50 to 80 min. Persons who took part in the qualitative interviews were rewarded with a duo cinema ticket. In line with guidelines for conducting qualitative research, focus groups were conducted until information saturation was reached [21]. This translates into one focus group for each province, except for East Flanders where two focus groups were conducted. In total, 31 people took part in six focus groups (Antwerp: city Wilrijk, *n* = 4; Limburg: township Alken, *n* = 4; East Flanders: city Ghent, *n* = 5 and *n* = 4; Flemish Brabant: township Boortmeerbeek, *n* = 1; West Flanders: township Desselgem, *n* = 13). In the township Boortmeerbeek only one person was present and an individual interview (approx. 20 min) was conducted.

### Quantitative measurements

At baseline, participants reported their demographics, general health and alcohol consumption (weekly alcohol use and binge drinking i.e. how often in the last six months a person drank four (for women)/ six (for men) or more glasses of alcohol within a two-hour period [22]) (supplementary file 2) [8], how they got to know TMC, their reason to participate and whether they participated individually or as team. During the post measurements, questions were asked about satisfaction with the timing of TMC and the campaign materials and whether participants of TMC had succeeded in achieving the one-month abstinence goal.

Participants could also indicate if, when and how much they drank alcohol during TMC, as well as the most difficult situations to refuse/drink less alcohol. In addition, participants were asked if they had changed other health behaviors during participating in TMC (e.g. drinking less/more soft drinks) and whether they experienced beneficial effects (e.g. saved money). They were also questioned about whether and how much more than usual they drank alcohol to reward themselves after refraining from alcohol for one month. Finally, participants were asked if they would participate in TMC again and why (not).

### Qualitative measurements

Participants who did not yet complete the baseline measurement (i.e., those reached through snowball sampling or via the master students) were first asked to complete a short demographic questionnaire (i.e. age, sex, education level, general health and alcohol use). Consecutively, based on a semi-structured interview guide, participants were asked about TMC reach and motivation to participate (e.g. ‘What were your motivations to participate in TMC?’), experiences (e.g. ‘How did you experience participating in TMC?’), perceived effectiveness (e.g. ‘Did you change any habits, besides alcohol use, during your participation?’) and perceived maintenance of the effect after TMC (e.g. ‘How was your alcohol consumption after participating in TMC?’). The full interview guide can be found in Supplementary File 3.

### Data analyses

Descriptive statistics (means with standard deviations and frequencies) were calculated using SPSS statistics software package version 25. Due to the non-random drop-out based on sex, age, educational level and work status (supplementary file 4), inverse probability weighting was applied on these variables [23, 24]. Quantitative descriptive analyses were conducted using survey weights calculated as the inverse of the propensity scores. The prevalences are based on the weighted data. There were some questions where logics were applied (e.g. only those who indicated that they did not wear the wristband were asked why they did not). To clarify this in the results, only when displaying data from a subsample, the numbers were added between brackets (“n=…”). The other results should be interpreted considering whether the question was asked at baseline (*n* = 49,022) or at post (*n* = 16,547).

The raw qualitative data, in particular the audio recordings of the focus groups, were transcribed verbatim, pseudonymized and entered into Nvivo 11 for analyses. A qualitative content analysis was conducted, a technique where categories present in textual data are identified following a systematic coding process [25]. All transcripts were first read several times to achieve immersion. Initially, categories were determined based on the key concepts of the interview guide and different (sub)categories were added based on the content of the data (see coding framework in Supplementary File 5). Subsequently, the coding process included combining codes that belonged together to reduce and categorize the data. Two researchers independently coded all interviews. Outputs were discussed, interpreted and summarized by the two researchers and an independent third researcher. For readability, sometimes the terms ‘some’ or ‘others’ are used to describe the qualitative data. However, in accordance with qualitative research [26, 27], no quantification is given. Results are presented according to the key elements of the process evaluation i.e. reach, experiences, perceived effectiveness and maintenance. For each element, qualitative and quantitative data were presented.

## Results

### Reach

TMC participants (i.e. those who filled in the quantitative questionnaires) and focus group participants were mainly women, higher educated, employed and had an average weekly alcohol consumption at baseline of 10.7 and 8.3 glasses respectively (Supplementary File 2). Characteristics of focus group participants were generally similar to those of the TMC participants, although there were more focus group participants with a high educational level and a high general health compared to the TMC participants (89.7% vs. 66.4% and 100% vs. 79.0% respectively) and weekly alcohol consumption was lower among focus group participants (8.3 vs. 10.7 glasses, respectively).

Participants got to know TMC mainly through social media (51.8%), television (39.7%) or through friends or family (27.1%). Main reasons to participate in TMC were ‘wanting to know how it physically or mentally would feel like to refrain from alcohol during one month’ (respectively 74.6% and 69.6% agreed or completely agreed). Several focus group participants saw participation as a challenge and wanted to find out whether they were able to say no to alcohol. Others indicated they already made plans to live healthier or had the intention to drink less alcohol. *[Woman, 50 years old about reason to participate in TMC]* “*I saw it as a challenge for myself, because I felt I needed a glass of wine every day, I thought, and then maybe a glass of beer in the evening. Sometimes it started with an aperitif when I was cooking. And I thought no, I want to get rid of that. I want to see if I can do it.”.*

Most participants (91.0%) aimed to totally refrain from alcohol during TMC, while some (9.0%) wanted to drink less alcohol. Most people (77.3%) participated in TMC on their own and 14.8% as part of a team. The focus groups revealed that participants were encouraged to participate after talking about TMC with family or friends or were stimulated to participate as a team through their work environment.

### Experiences

#### Timing of TMC

Almost all participants (93.6%) were satisfied with the month February as timing for TMC. Most participants (77.3%) complied with the proposed dates of TMC (i.e. 1–28 February). During the focus groups, participants stated that February is the ideal month to fulfil New Year’s resolutions and to ‘detox’ after a month with many festivities. Some focus group participants suggested that the campaign could be held twice a year (e.g. after New Year and after the summer months). Others thought one time a year was sufficient because otherwise it would not be ‘special’ anymore.

#### Campaign materials

Most campaign materials of TMC were considered useful and/or motivating (Supplementary File 6). Three quarter (73.3%) of the participants were satisfied with the number of received e-mails. The *weekly motivational e-mail* was read in detail by 55.0% of the participants. Focus group participants explained that the motivational emails were perceived as positive because they helped them to maintain the abstinence behavior, were a confirmation that they were doing well and included suggestions for non-alcoholic recipes. However, one participant indicated that the content of the e-mails could be more attractive, and another participant suggested to include a ‘tip of the day’. The *TMC website* was evaluated positively by most focus group participants: they found it professional, hip, fun and welcoming. They liked that different age categories were represented (e.g. the ‘grandmother’ in the promotional video). Some participants suggested the website could vary more over time e.g. a daily non-alcohol drink. The *online TMC badges* were found silly or not appealing to some focus group participants, while others were positive about them. The *campaign symbol of TMC*, a droplet, often appeared on Instagram which was experienced as positive. During TMC (a limited number of) *wristbands* and *pins* were distributed. Most participants did not see the wristband (90.2%) or pin (93.0%). Of those who did not see one (n = 2,350) respectively 61.8% and 46.5% indicated they would have liked to receive one (see Supplementary File 7 for reasons to (not) wear a wristband/pin). *[Woman, 32 years old about not wearing a wristband or pin during TMC].”Someone asks ‘Do you need something to drink’ and you say: ‘No, because I participate in TMC’. Everyone knew the concept. That was so widely known. So, I didn’t really think it was necessary to have a band or a pin as everyone knows what you’re doing. Everyone knew that.”.* Some participants indicated some disadvantages of the wristband (e.g. not a durable material). About the TMC pin, one focus group participant found it something strange, another thought wearing a pin provided a feeling of connection because friends and colleagues also wore it. [Woman, 30 years old about wearing a pin] *“I also found it [wearing a pin] a support for me to remind myself of it every day: I am committed to that [abstaining]. And I also found it helpful that other people saw that I was involved. […] Yes, I wore it with pride. The wristband, I have very thin wrists so that was not practical, it fell out all the time. I thought that pin, that’s easy, that’s visible. That is psychological for me, I still participate in it, so I liked that pin, yes.”.* One participant indicated that if it would have been possible to buy the wristband or pin to fund cancer research (one of the aims of TMC), he would have done this.

Participants indicated they lacked a forum or a space to talk with other participants about their (weekly) experiences. *[Woman, 46 years old about the (lack of a forum on) TMC website] “I think everyone should be able to talk about those moments [difficult moments during TMC]. There was also something [on the TMC website] about tips for difficult moments and they [organizers of TMC] should put some things in there, such as experiences of people who are participating: ‘I was at a cafe with friends and it has been very difficult [not to drink].’ Then you have the feeling that others also have difficult moments and you can learn how to deal with for example social pressure.“.* Several focus group participants felt the need for a TMC app. When focus group participants were asked to rate the campaign, in general, participation in TMC was experienced positive. Next, participants could give suggestions for improvement or could explain what they particularly liked about the campaign.

#### Facilitators during TMC

Several factors facilitated refraining from alcohol. Most friends and family of participants were aware of TMC (84.1%), did not criticize participation (62.3%) and 39.0% of participants were encouraged to participate in TMC (Table 1).

**Table 1:** Social environment, difficulties for drinking less or abstaining during TMC and perceived benefits of participation in TMC (*n* = 15,769)

Social environment (missing)	Completely disagree or disagree	Sometimes agree	Completely agree or agree
When I explained to my friends and/or family about my participation in TMC, the majority knew the campaign	4.9%	10.9%	84.1%
During my participation in TMC, I had more conversations about alcohol with friends and/or family than I normally had	24.5%	20.3%	55.2%
My friends and/or family have encouraged me to continue my participation in TMC	29.1%	31.9%	39.0%
My friends and/or family criticized my participation in TMC	62.3%	25.9%	11.8%
It was difficult to drink less or refrain from alcohol…			
…during social events (e.g. receptions, family dinners, parties)	28.9%	25.9%	45.2%
…because it was difficult to change certain habit(s) (e.g. glass of wine during the meal,…)	42.8%	20.4%	36.8%
…because it was difficult to find tasty non-alcohol alternatives at café or restaurant	47.4%	19.6%	32.9%
…because I like the taste	42.5%	26.8%	30.7%
…because I enjoy drinking alcohol	49.4%	24.2%	26.4%
…because alcohol was offered to me	49.2%	23.4%	27.5%
…because I had to deal with stress or wanted to relax	60.8%	17.3%	21.9%
…because I didn’t get support from my friends/family	79.0%	12.9%	8.1%
Perceived benefits of participation in TMC			
I was more aware of my usual alcohol consumption	8.4%	9.7%	82.0%
I felt better about myself	29.8%	30.0%	40.2%
I slept better	29.0%	31.6%	39.5%
I had more energy during my free time	32.4%	30.3%	37.4%
My general health was improved	32.8%	33.4%	33.9%
I ate healthier	40.2%	30.9%	28.9%
I was more productive (at work)	44.6%	31.8%	23.6%
I exercised more	56.5%	23.6%	19.9%
I had a more beautiful and smoother skin	52.2%	29.5%	18.2%
I was more pleasant to deal with	49.5%	33.6%	16.9%
I had more time for leisure activities/hobbies	60.0%	26.1%	13.9%

A first facilitator mentioned during the focus groups was *‘stimulus control*’ (i.e. removing cues for unhealthy habits and adding prompts for healthier alternatives). To make it easier for themselves, some participants spontaneously had removed all alcoholic drinks from their homes before the start of the campaign and made sure there was no alcohol in house during their participation. A second facilitator was *‘connectedness’*. Participants experienced TMC as a widespread campaign: TMC was mentioned in the newspaper, discussed in TV programs and on the radio, it was talked about at work and when going out. The campaign was alive and the hype created a sense of togetherness. Also, the *‘social environment’* of participants played an important role as facilitator, although sometimes it was also an obstacle (see further). Some participants shared their TMC participation on social media or informed friends/family. Participants indicated this gave them support and stimulated them. *[Woman, 54 years old about how social support helped during TMC]. “It also helps if… yes once people know, that supports you to continue, because they know you’re in on it. So they will automatically say ‘it’s right you’re not drinking this month’. They support you in that. If you did drink, they would say, ‘Have you failed?‘, ‘Have you given up?’ That gives you a bit of support to continue.”.*

Finally, it was also mentioned that not drinking eventually also became a facilitator for not drinking: *[Woman, 30 years old on how drinking during TMC became easier]. “I found the first two weeks especially difficult. Especially the first week because you are so very aware of: I can’t do it, I can’t. And then the weeks after that it went easier and easier and I was actually not so busy thinking about it anymore and it was almost obvious that I didn’t drink anything. At first it was like: “Ah I’m going to have a drink with a friend, oh no it can’t be wine!” later it came spontaneously: “ah I’m taking part in TMC, so a glass of coke”. It got better and better as the month went on, I noticed. That it got easier, yes.”*.

#### Obstacles during TMC

Participants experienced the most difficulties for abstaining during social occasions (45.2%) and when they had to change a habit (36.8%) (Table 1).

A first obstacle that was discussed during the focus groups was *breaking the drinking habit*. Several focus group participants indicated that it was mainly difficult to refrain from alcohol during weekends (e.g. a drink during social activities), while other participants found it difficult to abstain during weekdays (e.g. a drink to relax after work or during a meal). *[Men, 55 years old about obstacles of not drinking]*. “*Yes, it [alcohol beverages] tastes good and sometimes you eat something and then you think ‘with a glass of white wine that would be much better than with a glass of sparkling water’, so I think that’s the hardest part. I associate wine with food. Beer in a café is no problem, you can easily leave it.”*

A second obstacle during TMC was the *social environment* of participants. Participants were told things like: ‘are you really crazy?‘, ‘are you one of those [TMC participants]’ or even ‘TMC wuss’. The social environment of some participants wondered whether the participant might drink too much or have an alcohol problem. A participant told how drinking alcohol is ingrained in our culture: drinking is social and not drinking is antisocial for some. Some participants indicated that the goal of the campaign was not enthusiastically received everywhere with statements such as: *‘Another thing you should participate in’* or *‘we are doing a Tournée Genérale [counter movement to drink more alcohol]’.* Finally, finding alternative beverages was an obstacle. Many participants indicated that there were very few non-alcoholic alternatives available at cafes/restaurants. Also during a reception it was difficult for participants to find good, tasty alternatives to the standard alcoholic beverages. Some participants indicated that they would expect at least one mocktail (i.e. non-alcohol cocktail). Most participants were quickly tired of some of the frequently offered alternatives, such as fruit juice. One participant found it difficult to pay for water and preferred to buy something with more fizz or more flavour. *[Woman, 32 years old about (lack of) non-alcoholic alternatives.]. “[…] the waiter stands there and asks what it might be for an aperitif. And we ask: ‘What do you have?‘. Hup, a laundry list of alcoholic drinks. We all happened to be taking part in TMC, except for two of that table. And you can already see that the waiter is like ‘yeah, that will be a nice evening here with that table’. <<sigh > > The following week you are in another restaurant and it is immediately clear from the blackboard: ‘TMC cocktail 7.50 euros’. So yeah, not cheap either. There were only 4 of us at the time. Two who participated, two who did not participate. And even those who did not participate, also tasted. I think that makes a big difference in how that cafe or restaurant approaches it.”*

### Perceived effectiveness

Perceived effects of participation in TMC on alcohol consumption.

The majority of TMC participants (86.5%) did not drink alcohol during the month February, 9.0% drank less and 4.5% did not succeed. Of those consuming alcohol during TMC (*n* = 2.846), about three-quarters (78.7%) of participants who drank alcohol reported drinking on four days or less and 32% reported drinking on only one day. Lastly, we added the denominators in the tables. Participants drank alcohol mainly in the third and fourth week of the campaign. Most (71.2%) drank on average one or two glasses on these days and only 2.9% drank more than four glasses. Refraining from alcohol for one month was perceived (very) easy for most participants (65.9%).

In the focus groups, most participants told that their TMC participation made them more aware of their alcohol consumption and had broken the habit of drinking alcohol (e.g., having an aperitif before dinner, a glass of wine or a beer while cooking/eating, when taking a bath, in front of the television or after a leisure time activity).

#### Perceived benefits of participating in TMC

Most frequently perceived benefits of not drinking alcohol were ‘feeling better about themselves’ (40.2%), ‘sleeping better’ (39.5%) and ‘having more energy during free time’ (Table 1). Also, 82.0% indicated to be more aware of their usual alcohol consumption. Participants also drank (a lot) more water (56.2%), mocktails (43.1%) and tea (28.8%), and ate more fruits and vegetables (21.3%) during TMC.

Focus group participants *felt (physically) better* by not drinking alcohol for a month, even those who did not expect this. Participants reported feeling fresher or less tired during TMC (i.e., being more alert and sharper of mind), which was experienced as pleasant. For example, a participating student indicated to have more energy after a night out when not consuming alcohol, which made studying or working for school more successful. Another participant indicated that he recovered faster when not consuming alcohol after a sports performance compared to when he drank beer afterwards. Another benefit that regularly came up during the focus group discussions was *sleep*: sleeping more deeply, better or falling asleep faster.

A participant realized that there are *alternatives to cope with negative emotions*. He gained insight into why he drank (frustration, pressure or stress) and because of his participation he no longer automatically reached for a glass of wine after an exhausting day, but tried to deal with these feelings in a different way, e.g., by exercising. Some participants also indicated that they had done more sports during their participation in TMC. They mentioned that if you are motivated to refraining from alcohol, you are motivated for several other health behaviors as well, e.g., one participant stopped smoking because of his participation in TMC. Other experienced benefits were: better fitness, better taste, more voluminous hair, less cracked tongue, better sexual experience and clearer skin.

Although many participants experienced positive effects it remains a personal experience. For example, one participant mentioned that against her expectations, participation in TMC had no positive effects except for drinking more water.

#### Perceived adverse effects of TMC

Some TMC participants compensated refraining from alcohol with unhealthy behaviors such as drinking more diet and sugared soft drinks (resp. 36.8% and 27.7%), using more stimulant medication (23.9%) or illegal drugs (23.9%) (supplementary file 8). More than one third (36.6%) went (a lot) less to a bar, restaurant or party during TMC.

Some focus group participants indicated deliberately avoiding social contacts or going to a bar in February because the temptation was too strong to drink. Other participants were tired sooner during a night out and wanted to go to sleep instead of ordering another glass. However, most participants had not adapted their social activities during their participation in TMC.

Participants mentioned some disadvantages of drinking non-alcoholic beverages. For example, some reported feeling bloated if they drank soft drinks and water all evening or were worried about gaining weight when drinking unhealthy alternatives containing a lot of sugar. Some participants indicated that participating in TMC does not save money as non-alcoholic cocktails were equally expensive. Some participants said it did not feel right not being able to drink just like their friends (who drank alcohol).

### Perceived maintenance of the effect after TMC

#### Alcohol consumption after TMC

In the first week after TMC, one out of five participants (21.2%) did not drink alcohol and most participants (93.0%) did not drank more than usual to reward themselves for abstaining during one month. Furthermore, participants indicated to drink a maximum of 2.6 ± 3.0 glasses within 24 h, with 76.6% drinking less than 3 glasses within 24 h in the first week after TMC.

Most focus group participants had chosen something tasty as their first alcoholic drink after TMC. Some participants followed the rule of 10 [Belgian guideline to drink a maximum of 10 standard glasses of alcohol per week] after TMC. One participant thought alcohol had a strange taste, another participant felt guilty about starting again and alternated a glass of wine with water. Most participants drank less (during the week), indicated that they knew their limits better and drank less on each occasion.

#### Willingness to participate again and suggestions for next edition

Most participants (85.7%) indicated they would (most likely) participate in TMC again next year. Those who would not or were not sure mentioned they had experienced too few benefits (53.5%) or participation was no longer necessary (41.7%) (Supplementary File 9).

During the focus groups, participants provided advice for future TMC campaigns. They recommended a stronger focus on the guideline to drink a maximum of ten glasses a week. This can be done, for example, by trying to drink a maximum of ten glasses in one month (e.g. February) as a new challenge. Some participants would have liked to see more scientific evidence of the physical effect of the campaign e.g. by taking blood samples. Additionally, some participants suggested a stronger focus on other health aspects during TMC: drink more water, eat more fruit, stop smoking or exercise more.

Another suggestion was to give the hotel and catering industry a ‘label’ if they served a minimum number of non-alcoholic beverages or to hang a banner at the entrance of participating TMC cafes (e.g. indicating there is an equal number of non-alcoholic and alcoholic choices). Another suggestion was to have ready-made non-alcoholic alternatives (e.g. with fruit) in grocery stores. Participants also indicated that the presentation of a drink is important, e.g. using a festive, beautiful glass. It is important to be able to toast with the non-alcoholic beverage, so the atmosphere and cosiness is not lost and someone who does not drink alcohol is also part of the festivities.

## Discussion

This paper describes a comprehensive process evaluation of TMC examining reach, experience, perceived effectiveness and maintenance. Moreover, subcomponents of TAC evaluations i.e. the channels to reach participants, representativeness of participants and reasons for not participating (*reach*); participants’ use and response to all intervention components and materials (*experiences*); perceived impact of the intervention (including both desired and adverse effects) (*effectiveness*); and perceived maintenance of behavior change after the intervention (*maintenance*). Investigating these key elements in a process evaluation helps us to better understand the intervention effects and can inform future TACs.

This study revealed that most participants participated in the challenge because they wanted to know how it felt to abstain for one month. Comparable results were found among Dry January participants (TAC in England). ‘Health reasons’ and ‘to take on the challenge’ were indicated as the main motives to participate in Dry January [17]. From the current study, we could not derive how many Belgian adults exactly knew the campaign and how many participated in total. One year after the first TMC (on which the current study focused), a market research firm (Indiville) estimated that 18% of Belgian adults participated in the second edition of TMC [28].

Overall, participants experienced and evaluated TMC as positive. Most participants indicated they would participate again in a next edition of TMC. Most campaign materials of TMC were found to be useful and motivating, especially the motivational weekly e-mails and website of TMC. A previous study showed that greater use of email support was correlated with completing the Dry January challenge [6]. In the TAC Dry January, the website and app were positively evaluated but participants would like it to be more customized to their needs for support [17]. In the current study, focus group participants also indicated the importance of support (e.g. need for a forum to share experiences). Other comparable TAC studies [17, 19] showed that campaign materials presenting the benefits of taking part in the challenge, stories from other participants and tips to resist cravings or temptations were seen as valuable. Future studies should investigate whether the effect of TMC is moderated by satisfaction with or exposure to campaign materials.

Most participants completed TMC without drinking (86.7%). Comparable to the Dry January study (62.4% reported staying dry) [4], TMC participants who did drink during the challenge only consumed a few alcoholic beverages. We identified several facilitators and barriers towards abstinence during TMC. First, the abstinence experience during the beginning of TMC was perceived to be a facilitator to continue this behavior. In-depth interviews with FebFast participants (TAC in Australia) also showed that participants perceived not drinking for a month to be a self-discovery as it stimulated to create new beliefs which, in turn, facilitated abstinence [18]. However, our quantitative process evaluation showed that participants who failed the challenge (and did drink during February) mainly did this in the third and fourth week of the campaign. Second, participants indicated that they removed cues during the challenge (e.g. removing alcoholic beverages at home) to help themselves to stay abstinent. This is in line with several studies showing the importance of contextual cues in setting (healthy) behaviors [29–31]. In line with previous findings, the social environment showed to be both a facilitator and a barrier during the abstinence challenge. Some studies show that positive social support is associated with changes in the desired behavior (i.e. less alcohol consumption) [32–34]. However, Dry January participants who registered with another person were not more likely to complete the abstinence challenge [35]. Research on Hello Sunday Morning (TAC in Australia) showed that negative reactions from others were a barrier in achieving temporary abstinence [7]. In addition, habits were found to pose a barrier towards abstinence: breaking habits such as drinking a glass of wine during the preparation of a meal was found to be difficult for participants. The aim of a TAC is to make people reflect on their drinking behavior by making them aware of their drinking context. This reflection on habit (which is an automatic behavioral response) [36] makes drinking alcohol again a conscious behavior. However, although perceived as difficult, many participants succeeded in breaking these habits as the effect evaluation of TMC showed a change in drinking habit after participation [8]. Finally, another barrier appeared to be finding worthy alternatives for alcohol beverages during the abstinence challenge. Health concerns (e.g. calorie uptake) regarding non-alcohol beverages have to be taken into account when promoting alternative non-alcohol beverages [37]. In addition, the price, availability and visibility of non-alcohol beverages is important to give and stimulate healthy choices (e.g. non-alcohol alternatives) [38, 39]. To conclude, the impact of perceived facilitators and obstacles during a TAC on (sustaining) the effect of the campaign should be further investigated to identify the main facilitators and obstacles future TACs should focus on.

Several perceived benefits of participating in TMC were observed: feeling better, sleeping better and having more energy during free time. Several studies [16, 17, 19] described similar benefits perceived by Dry January participants: improved physical and psychological wellbeing, sleep quality, concentration, energy levels, skin, losing weight and saving money. An added value of our study was that, perceived adverse effects of participation were also investigated. There was a possible economic impact of the campaign (e.g. one third went (a lot) less to a bar, restaurant or party during TMC). In addition, some participants compensated refraining from alcohol with unhealthy behavior (e.g. drinking more sugared soft drinks or using more drugs). When alcohol consumption is used as coping mechanism (e.g. in case of stress) and this use is discontinued, it is possible that other substances are used [40, 41]. To limit the consumption of sugared non-alcoholic beverages and drugs and to avoid a decrease in spending’s, future TACs could promote healthy coping mechanisms to deal with stress (e.g. performing physical activity) and could work together with the hospitality sector to provide a wider range of healthy and attractive alternatives.

Most participants indicated to know the campaign through mass media channels (e.g. social media, television) or through family or friends and we can tentatively say that TMC was a widespread, well-known campaign in Belgium. Therefore, future TACs may also use these channels to disseminate their campaign to the general public. We also advise to keep the campaign message light e.g. by focusing on “doing a challenge” as this was perceived as positive (i.e. not pointing fingers about drinking too much). Research on two Australian TACs found that the effectiveness is greater when participants are portrayed positively (e.g. hero, fundraiser) than negatively (e.g. being a drinker or must do a detox) [42]. Since social support appears to be an important factor, future TACs are advised to focus on the social environment of participants by, for example, strongly emphasizing on a sense of belonging to a group or trying to create some sort of ‘we are in this together’ feeling. This can be done by using social media channels (e.g. encourage participants to share messages of their participation) or providing a platform on the campaign website where participants can find and interact with like-minded people. Providing participants with knowledge and skills on how to break habits seems important, but developers are advised to be aware that participants often only become aware of their habits by participating in the campaign. To break alcohol habits, several tips can be given to participants such as removing cues (e.g. no alcohol beverages at home) or thinking about or making arrangements around drinking in advance (e.g. providing non-alcoholic alternatives when hosting a home party). Finally, the accessibility, attractiveness and healthiness of non-alcoholic beverages should be improved. This recommendation will require collaboration with the hospitality sector and a general shift in perspective in our ‘pouring out’ habits.

A strength of this study was the use of a mixed-methods approach: combining quantitative and qualitative data increases the integrity and applicability of findings [43]. The qualitative data (1) confirmed some of the quantitative findings (e.g. almost all participants were satisfied with the month of February (quantitative data)), (2) gave more in-depth insight (e.g. gives some reasons why participants liked the month February (qualitative data)) or (3) refuted some of the quantitative findings (e.g. more than one third went (a lot) less to a bar or restaurant during TMC (quantitative data), while most focus group participants did not adapt their social activities (qualitative data)). Another strength was that important indicators (e.g. reach, experiences, effectiveness and maintenance) for process evaluation research were examined [44]. However, this resulted in recommendations that can only improve participants’ experiences. Future research should also assess the experiences of adopters and/or implementers of TACs. Assessing their needs and experiences could improve future (implementation of) TACs and possibly even the impact on TAC participants.

Although this study provided valuable insights into the process of TMC, there were also some limitations. TMC participants were self-selected resulting in a non-representative sample of the Belgian population. Compared to the general Belgian population, TMC participants in the present study were older, more likely to be female and higher educated, but had a similar weekly alcohol consumption [8]. These socio-demographic differences limit the generalizability of the findings of the present study to the general Belgian population. In addition, we should take into account that not all TMC participants took part in the current study. Therefore, it is possible that TMC participants included in this study were more willing to change their alcohol consumption and were more convinced about TMC (materials), leading to a more positive evaluation of TMC. This participation bias is not uncommon in (TAC) research [4, 45]. In addition, the characteristics of focus group participants differed from those who took part in the questionnaires. However, the purpose of the focus groups was mainly to gain in-depth insights and not to recruit a representative sample. Nevertheless, the results should be viewed critically since some subgroups were underrepresented in the focus groups (e.g., the less educated, those with a lower general health status and heavy drinkers) who could have had different opinions on TMC (materials). Another limitation was the non-random drop-out between complete and non-complete cases. We used inverse probability weighting to correct for the non-random drop-out. However, weighting can only take into account the variables that were measured [46], so it is possible that, despite this correction, there is still an over- or underestimation of certain subgroups of TMC participants. Another limitation is the possibility of socially desirable answers [47]. Finally, this study was conducted in Belgium which consists of different language communities (Dutch-, French-, and German-speaking) [48]. Although TMC was a nationwide campaign, focus groups were only conducted with Dutch speaking participants and we could not take into account any cultural differences between different country regions.

## Conclusion

In conclusion, TMC was experienced positively by participants. Future TACs could use its methods and further improve it by including for example a social support network during the campaign (e.g. forum to share experiences) or collaborate with the hospitality sector to create a shift on the presentation and availability of non-alcoholic beverages.

### Electronic supplementary material

Below is the link to the electronic supplementary material.

Supplementary Material 1

## Data Availability

The datasets used during the current study are available from the corresponding author on request.

## Abbreviations

TAC: Temporary Abstinence Campaign
TMC: Tournée Minérale campaign

## References

[1] WHO. Global status report on alcohol and health 2018. World Health Organization; 2019. p. 9241565632. Report No.

[2] Brown C, Stewart SH (2021). Harm reduction for women in treatment for alcohol use problems: exploring the impact of dominant addiction discourse. Qual Health Res.

[3] Reynolds J, McGrath M, Engen J, Pashmi G, Andrews M, Lim J (2018). Processes, practices and influence: a mixed methods study of public health contributions to alcohol licensing in local government. BMC Public Health.

[4] de Visser RO, Piper R (2020). Short-and longer-term benefits of temporary alcohol abstinence during ‘Dry January’are not also observed among adult drinkers in the general population: prospective cohort study. Alcohol Alcohol.

[5] Hillgrove T, Thomson L (2012). Evaluation of the impact of FebFast participation (VicHealth).

[6] de Visser RO, Nicholls J (2020). Temporary abstinence during dry January: predictors of success; impact on well-being and self-efficacy. Psychol Health.

[7] Pennay A, MacLean S, Rankin G, O’Rourke S (2018). Hello Sunday Morning: strategies used to support temporary alcohol abstinence through participation in an online health promotion program. Health Promotion J Australia.

[8] Thienpondt AVC, Van Jelle J, Nagelhout G, Deforche. Benedicte. Effect evaluation of the one-month-abstinence campaign ‘Tournée Minérale’ on alcohol consumption in Belgian adults. 10.1186/s13690-024-01491-2 PMC11795991 39910641

[9] Grant A, Treweek S, Dreischulte T, Foy R, Guthrie B (2013). Process evaluations for cluster-randomised trials of complex interventions: a proposed framework for design and reporting. Trials.

[10] McGill E, Marks D, Er V, Penney T, Petticrew M, Egan M (2020). Qualitative process evaluation from a complex systems perspective: a systematic review and framework for public health evaluators. PLoS Med.

[11] Linnan L, Steckler A. Process evaluation for public health interventions and research. 2002.

[12] Lockwood I, Walker RM, Latimer S, Chaboyer W, Cooke M, Gillespie BM (2022). Process evaluations undertaken alongside randomised controlled trials in the hospital setting: a scoping review. Contemp Clin Trials Commun.

[13] Moore GF, Audrey S, Barker M, Bond L, Bonell C, Hardeman W et al. Process evaluation of complex interventions: medical research council guidance. BMJ. 2015;350. 10.1136/bmj.h1258 PMC4366184 25791983

[14] Saunders RP, Evans MH, Joshi P (2005). Developing a process-evaluation plan for assessing health promotion program implementation: a how-to guide. Health Promot Pract.

[15] Glasgow RE, Harden SM, Gaglio B, Rabin B, Smith ML, Porter GC (2019). RE-AIM planning and evaluation framework: adapting to new science and practice with a 20-year review. Front Public Health.

[16] de Visser R. Young people and temporary alcohol abstinence during dry january. Young adult drinking styles: current perspectives on research, policy and practice. 2019:253–72.

[17] de Visser R, Lockwood N (2019). Evaluation of dry January 2019.

[18] Robert J (2018). Meeting the sober self, recognizing the drinking self: back to baseline experimentation in temporary sobriety initiatives. Contemp Drug Probl.

[19] Yeomans H (2019). New Year, New You: a qualitative study of dry January, self-formation and positive regulation. Drugs: Educ Prev Policy.

[20] Sahin MD, Öztürk G (2019). Mixed Method Research: theoretical foundations, designs and its use in Educational Research. Int J Contemp Educational Res.

[21] Morgan DL, Krueger RA, King JA. The focus group guidebook: Sage; 1998.

[22] NIAAA. Drinking levels defined: national institute on alcohol abuse and alcoholism; 2023 [Available from: https://www.niaaa.nih.gov/alcohol-health/overview-alcohol-consumption/moderate-binge-drinking

[23] Chesnaye NC, Stel VS, Tripepi G, Dekker FW, Fu EL, Zoccali C (2022). An introduction to inverse probability of treatment weighting in observational research. Clin Kidney J.

[24] Seaman SR, White IR (2013). Review of inverse probability weighting for dealing with missing data. Stat Methods Med Res.

[25] Hsieh H-F, Shannon SE (2005). Three approaches to qualitative content analysis. Qual Health Res.

[26] Busetto L, Wick W, Gumbinger C (2020). How to use and assess qualitative research methods. Neurol Res Pract.

[27] Nyumba O, Wilson T, Derrick K, Mukherjee CJ (2018). The use of focus group discussion methodology: insights from two decades of application in conservation. Methods Ecol Evol.

[28] VAD. Bijna één Belg op vijf nam deel aan Tournée Minérale 2018 - Ook in 2019 februari zonder alcohol; 2018 [Available from: https://www.vad.be/artikels/detail/bijna-een-belg-op-vijf-nam-deel-aan-tournee-minerale-2018---ook-in-2019-februari-zonder-alcohol

[29] Hagger MS, Lonsdale A, Koka A, Hein V, Pasi H, Lintunen T (2012). An intervention to reduce alcohol consumption in undergraduate students using implementation intentions and mental simulations: a cross-national study. Int J Behav Med.

[30] Qureshi A, Monk R, Pennington C, Li X, Leatherbarrow T, Oulton JR (2021). Visual and auditory contextual cues differentially influence alcohol-related inhibitory control. Adicciones.

[31] Rose AK, Brown K, MacKillop J, Field M, Hogarth L (2018). Alcohol devaluation has dissociable effects on distinct components of alcohol behaviour. Psychopharmacology.

[32] Lasimbang HB, Shoesmith W, Mohd Daud MNB, Kaur N, Jin MCP, Singh J (2017). Private troubles to public issue: empowering communities to reduce alcohol-related harm in Sabah. Malaysia Health Promotion Int.

[33] Lechner WV, Laurene KR, Patel S, Anderson M, Grega C, Kenne DR (2020). Changes in alcohol use as a function of psychological distress and social support following COVID-19 related University closings. Addict Behav.

[34] Liamputtong P, Koh L, Wollersheim D, Walker R (2015). Peer support groups, mobile phones and refugee women in Melbourne. Health Promot Int.

[35] de Visser RO, Robinson E, Bond R (2016). Voluntary temporary abstinence from alcohol during dry January and subsequent alcohol use. Health Psychol.

[36] Albery IP, Collins I, Moss AC, Frings D, Spada MM (2015). Habit predicts in-the-moment alcohol consumption. Addict Behav.

[37] Sikalidis AK, Kelleher AH, Maykish A, Kristo AS (2020). Non-alcoholic beverages, old and novel, and their potential effects on human health, with a focus on hydration and cardiometabolic health. Medicina.

[38] Quirmbach D, Cornelsen L, Jebb SA, Marteau T, Smith R (2018). Effect of increasing the price of sugar-sweetened beverages on alcoholic beverage purchases: an economic analysis of sales data. J Epidemiol Community Health.

[39] Whelan J, Millar L, Bell C, Russell C, Grainger F, Allender S (2018). You can’t find healthy food in the bush: poor accessibility, availability and adequacy of food in rural Australia. Int J Environ Res Public Health.

[40] Bendau A, Viohl L, Petzold MB, Helbig J, Reiche S, Marek R (2022). No party, no drugs? Use of stimulants, dissociative drugs, and GHB/GBL during the early COVID-19 pandemic. Int J Drug Policy.

[41] Smart JA. The relationship between social skills and coping drinking motives: a test of the social skills deficit vulnerability model. Utah State University; 2021.

[42] Bartram A, Hanson-Easey S, Eliott J (2018). Heroic journeys through sobriety: how temporary alcohol abstinence campaigns portray participant experiences. Int J Drug Policy.

[43] Schifferdecker KE, Reed VA (2009). Using mixed methods research in medical education: basic guidelines for researchers. Med Educ.

[44] Steckler AE, Linnan LE. Process evaluation for public health interventions and research. Jossey-Bass/Wiley; 2002.

[45] Elston DM. Participation bias, self-selection bias, and response bias. J Am Acad Dermatol. 2021. 10.1016/j.jaad.2021.06.025 34153389

[46] De Leeuw ED, Hox J, Dillman D. International handbook of survey methodology. Routledge; 2012.

[47] Bergen N, Labonté R (2020). Everything is perfect, and we have no problems: detecting and limiting social desirability bias in qualitative research. Qual Health Res.

[48] Mettewie L, Mensel LV (2023). Understanding foreign language education and bilingual education in Belgium: a (surreal) piece of cake. Int J Bilingual Educ Biling.

