# Supplementary material for: Process evaluation of the Belgian one-month-without alcohol campaign ‘Tournée Minérale’: a mixed method approach
Source: BMC Public Health. 2024 Feb 5;24:383. doi: 10.1186/s12889-024-17941-z (PMC10840226; doi:10.1186/s12889-024-17941-z)
Supplement: Supplementary file 1 — Supplementary Material 1 [file 12889_2024_17941_MOESM1_ESM.docx]

**Process evaluation of the Belgian one-month-without alcohol campaign ‘Tournée Minérale’: a mixed method approach.**

**SUPPLEMENTARY FILES**

Supplementary File 1: campaign materials of Tournée Minérale: practical application and channel(s)

| Practical application | Channel(s) |
| --- | --- |
| Participants were challenged to abstain from alcohol for one month | Website  Social Media  TV-spot |
| Participants could register on TMC website (as individual or in group) and share the participation online | Website  Social Media |
| An overview of possible health benefits of not drinking alcohol (feeling fitter, having a better skin, sleeping better, losing weight, saving money and not having a hangover) were shared | Website  E-mail  Social media |
| An overview of alternatives for alcoholic beverages (such as Mocktails) were shared | Website  E-mail  Social media |
| Participants could calculate how much weight they could lose and money they could save with refraining from alcohol with the ‘Alcohol calculator’ | Website  Social media |
| Good practice stories (other persons telling how to break drinking habits) and information about habitual drinking were shared | Website  E-mail  Social Media |
| Participants could weekly claim an online sober ‘badge’ for completing the abstinence challenge (four badges in total) | Social Media  E-mail |
| Social events (e.g. parties) could indicate that they support TMC | Social media |
| Wristband and pins of TMC were distributed | Intermediaries |
| Participants weekly received motivational messages to complete the abstinence challenge | Email  Social media |

Supplementary File 2: Baseline characteristics of TMC participants and focus group participants

| **Variable** | **Question** | | **Answer options** | | | | **Recoding** |  |
| --- | --- | --- | --- | --- | --- | --- | --- | --- |
| Sex | Are you…? | | 0 = Male; 1 = Female | | | |  |  |
| Age | What is your birthday? | | Dd/Mm/Yy | | | | … years old  0= younger (18–43 years old)  1= older (44-95 years old), based on the mean age of the sample |  |
| Education level | What is your highest degree? | | No degree (1); Primary education (2); Secondary education (3); Higher college education (4); Higher university education (5) | | | | 1 = Lower educated (1, 2 or 3); 2 = Higher educated (4 or 5) |  |
| Work status | What is your current work status? | | Student (1); Retired (2);  Housewife/man (3); Unemployed (4); Employed (5) | | | | 1= not working, 2= working |  |
| General health | How is your state of health in general? | | From very bad (1) to very good (5) | | | | 1=Low general health (1, 2 or 3); 2=high general health (4 or 5) |  |
| Baseline characteristics | | TMC participants (n=49.022)  % or M ± SD (missing) | | Focus group participants (n=31) | | | | |
|  | |  |  | % or M ± SD (missing) | | | | |
| Age  Men  High education level  Employed  High general health  Weekly alcohol consumption  Binge drinkers | | 44.5 ± 13.1 (1.074)  37.0% (3.923)  66.4% (699)  80.6% (699)  79.0% (761)  10.7 ± 13.8 (711)  63.5% (1.752) | | |  | 47.3 ± 14.3 (0)  33.3% (1)  89.7% (2)  85.7% (3)  100% (4)  8.3 ± 6.6 (6)  57.7% (5) | | |

M= mean; SD= standard deviation

Supplementary File 3: interview guide

1. How was your experience with Tournée Minérale?

- Can you describe some positive experiences with Tournée Minérale?
- Can you describe some negative experiences with Tournée Minérale?
- If you had to award a grade out of ten, how much would you award the campaign?
  - Why not a lower grade OR why not a higher grade?
- Tournée Minérale was about drinking no or less alcohol for one month. What was it like for you? Did you succeed in Tournée Minérale?

1. Are there certain things that helped you to drink no or less alcohol during Tournée Minérale? Can you tell us more about this?
2. Different materials were provided for the campaign *(examples are shown on a paper*), how did you experience these campaign materials?

- Are there any other elements you think of that could have been offered during Tournée Minérale?

1. Did you experience difficulties in drinking less or no alcohol during Tournée Minérale? Can you explain this?

- Are there any campaign materials that have helped with your difficulties? How did these campaign materials help with your difficulties?

1. Did you change other habits besides drinking alcohol during your participation? *For example: some people who start exercising more, also start eating more.*

- Can you say something about your social life during Tournée Minérale?

1. You participated in Tournée Minérale in February. What impact, if any, did your participation in Tournée Minérale had on yourself shortly after your participation?

- How was your alcohol consumption shortly after Tournée Minérale?
- How do you feel about your alcohol consumption now compared to before your participation?
- What are your expectations concerning your alcohol consumption in the future?

1. How has your participation in Tournée Minérale affected your friends and/or family?

- How was the alcohol consumption among your friends and/or family shortly after Tournée Minérale?
- How do you feel about the alcohol consumption of your friends and/or family now?

1. If Tournée Minérale were to be organized again next year, how would you handle it?

- What do you think the campaign should focus on next year?
- How would the campaign help you participate next year?
- How did you handled your participation now, how would you do it next year?
- Only if they answer "I won't participate next year anyway”:
  - Why will you not participate next year? What did you find difficult or inconvenient now?
  - Are there things that would make you want to participate again?
  - Are there materials or other things that might convince you to participate again, and what are they?

1. From what was said, what is the most important to you? What do you think I should definitely remember from this conversation?
2. Is there anything else you would like to share?

Supplementary File 4: Characteristics and drop-out analyses completers (baseline and post) vs. non-completers (only baseline).

| Baseline characteristics (n=missings) | Completed baseline and post (n=15,769) | Only completed baseline (n=32,481) | Comparison | |
| --- | --- | --- | --- | --- |
|  | M ± SD or % | M ± SD or % | t or chi² | p |
| % Men | 38.0% | 36.5% | 10.1 | **0.002** |
| Age (M ± SD) | 47.2 ± 13.0 | 43.1 ± 12.9 | t=32.0 | **<0.001** |
| % high education level | 69.7% | 64.5% | 130.2 | **<0.001** |
| % working | 78.2% | 81.8% | 85.9 | **<0.001** |
| Weekly alcohol consumption (M ± SD) | 10.9 ± 13.8 | 10.6 ± 14.0 | t=-1.8 | 0.073 |

M= mean; SD= standard deviation; significant p-values in bold

Supplementary file 5: Coding framework of qualitative data analysis

| **Category** | **Subcategory** | **Code** | **Description** |
| --- | --- | --- | --- |
| Reach | Channels to reach participants | Channel | Ways in which participants came in touch with TMC |
|  |  | Contact | How participant came in contact with TMC |
|  | Representativeness of participants | Awareness of TMC | The extent to which the campaign was known to the general public |
|  |  | Alcohol | Attitude, opinion and knowledge on alcohol and its consequences |
|  | Motivations for (not) participating | Reasons to consume alcohol | Reasons to consume alcohol before TMC |
|  |  | Alcohol use before TMC | Perception of their own alcohol use before TMC |
|  | Reasons for (not) participating | General reasons to participate | General reasons participants cited to participate in TMC |
|  |  | Social reasons to participate | Participating in TMC because of social reasons |
|  |  | Research reasons to participate | Participating in TMC to support scientific research |
|  |  | Health reasons to participate | Participating in TMC to improve health |
|  |  | Reasons not to participate | Reasons participants got from their family and friends not to  participate |
| Experiences | Timing of TMC | Timing / month February | Experiences concerning the month when TMC was implemented |
|  | Participants’ use of TMC (materials) | Use of TMC materials | The use of TMC materials |
|  |  | Banner | All thoughts concerning TMC’s banner |
|  |  | Facebook | All thoughts concerning TMC’s social media channel |
|  |  | Mails | All thoughts concerning TMC’s motivation e-mails |
|  |  | Pin | All thoughts concerning TMC’s pin |
|  |  | Wristband | All thoughts concerning TMC’s wristband |
|  |  | Website | All thoughts concerning TMC’s website |
|  |  | Advertisement | All thoughts concerning TMC’s advertisement |
|  | Participants’ response to TMC (materials) | Non-alcohol alternatives: attitudes & opinions | Attitudes and opinions towards non-alcoholic alternatives |
|  |  | Non-alcohol alternatives: disadvantages | Disadvantages of non-alcohol alternatives |
|  |  | Media | The way (social) media helped during TMC |
|  |  |  |  |
|  |  | Non-alcohol alternatives | Opinions and use of non-alcohol alternatives during TMC |
|  |  | Emotions during TMC | Experiences and feelings during TMC |
|  | Facilitators during TMC | Coping with difficulties during TMC | Strategies to cope with difficult situations during TMC |
|  |  | Resources during TMC | Perceived tools that facilitated their participation |
|  |  | Social control/support | The way social support helped during TMC |
|  |  | Alcohol experiences | Experiences where alcohol has (not) been refused |
|  | Obstacles during TMC | Difficulties during TMC | Perceived obstacles during their participation |
|  |  | Lack of alternatives | The (lack of) alternatives for alcohol beverages during TMC |
|  |  | Lack of social support | Lack of social support during TMC |
|  |  | Social pressure | Perceived pressure of others to drink alcohol during TMC |
|  |  | Habits | Routines or habits of drinking alcohol |
| Effectiveness | Perceived effectiveness | Ability to persevere | Believe in their own ability to persevere |
|  |  | Awareness | Changes in level of awareness about alcohol |
|  |  | Effect on alcohol | Changes in drinking behavior due to participation in TMC |
|  | Perceived impact of TMC: desired effects | Physical changes | Physical changes as result of TMC participation |
|  |  | Emotional changes | Mental changes as a results of TMC participation |
|  |  | Desired changes in behavior (not alcohol) | Desired behavior changes besides alcohol during and after TMC |
|  | Perceived impact of TMC: adverse effects | No impact of TMC participation | Perceived lack of impact of TMC |
|  |  | Avoiding social contact | Not doing social activities during TMC |
|  |  | Perceived adverse effects of TMC | Perceived adverse effects of TMC |
| Maintenance | Perceived maintenance of behavior change after TMC | Alcohol consumption after TMC | Perceived alcohol consumption after TMC |
|  | Next edition | Organizing TMC again | Thoughts on a next edition of TMC |
|  |  | Participating again | If en why (not) participating again next edition of TMC |
|  |  | Suggestions to improve next TMC | All suggestions to improve the next edition of TMC |

Supplementary File 6: Campaign materials: usefulness, motivational and thoroughness of reading (n=15,769)

| useful and/or motivating | Did not see this material | Completely disagree or disagree | Sometimes agree | Completely agree or agree |
| --- | --- | --- | --- | --- |
| Motivational e-mails | 2.9% | 9.5% | 12.9% | 74.8% |
| Website | 13.0% | 13.5% | 16.8% | 56.7% |
| Facebook | 31.0% | 13.2% | 10.6% | 45.1% |
| Campaign movie | 28.1% | 13.4% | 11.9% | 46.6% |
| Flyers | 60.7% | 18.7% | 7.3% | 13.2% |
| Thoroughness | Did not see this material | Have read (almost) nothing | Have read some pieces in detail, others only briefly | Have read (almost) everything in detail |
| Motivational e-mails | 2.3% | 19.5% | 23.1% | 55.0% |
| Website | 7.6% | 33.0% | 30.5% | 28.8% |
| Facebook | 24.4% | 34.4% | 20.2% | 21.0% |

Supplementary File 7: wristbands and pins: usefulness and reasons why owners did not wear it

| Usefulness of wearing | Carriers of a wristband (n=432) | Carriers of a pin (n=269) |
| --- | --- | --- |
| It made my participation easier to discuss | 275 | 162 |
| It reminded me not to drink alcohol | 130 | 45 |
| It made me less justify to others why I didn't want to drink alcohol | 168 | 110 |
| Reason not to wear wristband/pin | Owners of wristband (n=243) | Owners of pin (n=211) |
| I didn't feel the need to use the wristband/pin to indicate that I was taking part in TMC | 140 | 100 |
| I don't like to wear wristband/pin | 92 | 100 |
| It was not possible to wear the wristband/pin because of work, sports, ... | 41 | 12 |
| The wristband/pin just didn't appeal to me | 29 | 19 |
| The wristband/pin did not match my outfit | 14 | 13 |
| I didn't wear the wristband/pin because I never drink anyway | 1 | 2 |
| I was the only one who had the wristband/pin | 7 | 7 |
| I was afraid of comments from my environment about the wristband/pin | 2 | 7 |
| I didn't like the colour of the wristband/pin | 3 | 1 |

Supplementary file 8: potential compensation behavior during TMC (n=15,769)

| Use during TMC | Never used | (a lot)  less used | Used as much | (a lot)  more used |
| --- | --- | --- | --- | --- |
| Water | 0.50% | 9.00% | 34.30% | 56.20% |
| Mocktails | 38.50% | 4.80% | 13.60% | 43.10% |
| Diet soft drink | 29.60% | 9.70% | 24.00% | 36.80% |
| Thee | 18.20% | 13.90% | 39.00% | 28.80% |
| Sugared soft drink | 37.90% | 13.10% | 22.30% | 27.70% |
| Stimulant medication (e.g. Relatine) | 71.70% | 1.40% | 2.90% | 23.90% |
| Other illegal drugs | 71.70% | 1.60% | 2.90% | 23.90% |
| Cannabis | 71.00% | 1.90% | 3.50% | 23.60% |
| Sweet snacks | 10.80% | 22.80% | 43.60% | 22.80% |
| Gambling | 69.80% | 2.70% | 4.80% | 22.80% |
| Sedative medication (e.g. Xanax, Valium) | 70.40% | 2.70% | 4.10% | 22.80% |
| Sleep medication (e.g. Zolpidem) | 68.50% | 3.40% | 5.60% | 22.60% |
| Cigarettes | 65.40% | 5.40% | 7.10% | 22.10% |
| Vegetables and fruit | 0.50% | 18.80% | 59.50% | 21.30% |
| Fruit juice | 30.50% | 14.60% | 34.60% | 20.30% |
| Coffee | 14.10% | 18.30% | 47.70% | 19.90% |
| Computer- or videogames | 55.90% | 6.90% | 17.40% | 19.80% |
| Fast-food | 33.80% | 17.90% | 32.00% | 16.30% |
| Salty snacks | 12.60% | 29.40% | 42.00% | 16.00% |
| Bar/restaurant or a party | 5.50% | 36.60% | 54.40% | 3.50% |

Supplementary File 9: reasons why not willing to participate again next edition of TMC (n=2.124)

| I experience few benefits from my participation | 53.5% |
| --- | --- |
| I don't think it's necessary (anymore) to participate again | 41.7% |
| There are (too) few non-alcoholic alternatives (e.g. for special occasions, at a café/restaurant, etc.) | 18.6% |
| I don't feel like it anymore | 14.6% |
| I found it too difficult | 8.7% |
| The month of February doesn't really suit me | 6.4% |
| I have received too much criticism from my environment | 4.3% |
